# Stabilization of phenanthrene anions in helium nanodroplets

**DOI:** 10.1039/d2cp00991a

**Published:** 2022-05-04

**Authors:** Siegfried Kollotzek, Farhad Izadi, Miriam Meyer, Stefan Bergmeister, Fabio Zappa, Stephan Denifl, Olof Echt, Paul Scheier, Elisabeth Gruber

**Affiliations:** Institut für Ionenphysik und Angewandte Physik, Universität Innsbruck A-6020 Innsbruck Austria E.Gruber@uibk.ac.at; Department of Physics, University of New Hampshire Durham NH 03824 USA

## Abstract

It has been debated for years if the polycyclic aromatic hydrocarbon phenanthrene exists in its anionic form, or, in other words, if its electron affinity (EA) is positive or negative. In this contribution we confirm that the bare phenanthrene anion Ph^−^ created in a binary collision with an electron at room temperature has a lifetime shorter than microseconds. However, the embedding of neutral phenanthrene molecules in negatively charged helium nanodroplets enables the formation of phenanthrene anions by charge transfer processes and the stabilization of the latter in the ultracold environment. Gentle shrinking of the helium matrix of phenanthrene-doped HNDs by collisions with helium gas makes the bare Ph^−^ visible by high-resolution mass spectrometry. From these and previous measurements we conclude, that the EA of phenanthrene is positive and smaller than 24.55 meV.

## Abstract 2

Phenanthrene anions are stabilized in the ultracold environment of helium nanodroplets. Gentle shrinking of the helium matrix by collisions with helium gas makes the bare phenanthrene anion visible by high-resolution mass spectrometry.

## Graphical abstract

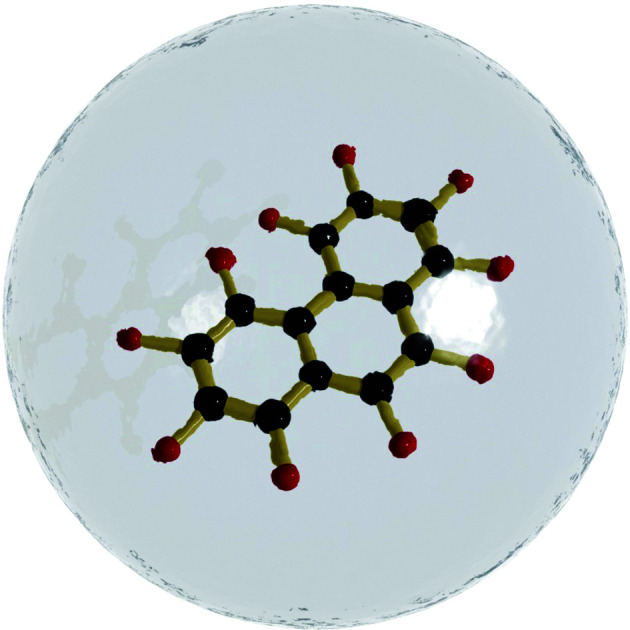

## Introduction

Polycyclic aromatic hydrocarbon (PAH) molecules are hydrocarbon compounds with multiple six-membered aromatic rings which are abundantly present in the interstellar medium in the form of neutral closed shell-molecules, molecular radicals, and ionized species. In the past few decades, anions of PAHs have received considerable attention as in regions with high electron densities and low UV photon flux, PAHs are considered to exist predominantly as negatively charged ions and influence substantially the chemical reactions in dense interstellar clouds.^1^ The stability of the anionic form relative to its neutral counterpart is governed by the electron affinity (EA). As a rule of thumb, an EA above 0.5 eV is needed for the formation of long-lived PAH anions, *i.e.* anions with lifetimes above 1 μs upon attachment of thermal electrons to PAHs.^2–5^ A negative EA means that the anionic form is not stable and isolated species of negative charge do not exist.

The EA of PAHs generally increases with increasing molecular size. Thus, benzene (C_6_H_6_) and naphthalene (C_10_H_8_) have a negative EA, while anthracene (C_14_H_10_, An) is the smallest unsubstituted PAH with a positive EA. A recent experimental value deduced from photoelectron spectroscopy (PES) of An^−^ showed an EA of 0.532(3) eV,^6^ in good agreement with earlier PES results^7–9^ and values obtained with other experimental approaches^10–12^ as well as theoretical calculations.^13–20^ A benchmark theoretical study accounting for zero-point vibrational energies and geometry relaxation effects results in an EA of 0.526(6) eV,^21^ matching the experimental value perfectly. An^−^ readily forms upon attachment of low-energy electrons to An molecules in the gas phase.^2,6–9,22,23^

The situation for phenanthrene (Ph), a bent isomer of An, is less satisfying. Several attempts to form the parent anion Ph^−^ by attaching low-energy electrons to Ph in the gas phase have failed so far,^2,22,24^ indicating that its EA is less than that of An. Several experimental studies using the electron-capture detector technique which is based on the temperature dependent kinetics of electron attachment have reported adiabatic electron affinities (AEAs) of Ph close to 0.3 eV.^10–12,25–27^ However, the validity of these data has been questioned,^5,14,15,28^ partly because the mass of the formed anions has not been measured, and partly because theoretical values for the AEA of Ph are well below 0.3 eV, or even negative.^13,17,29,30^ A benchmark study that includes structural relaxation and zero-point vibrational energy corrections places the AEA of Ph at −0.08 eV.^29^ To our knowledge, so far, only Lee *et al.* reported the observation of a small signal due to the parent anion, Ph^−^, by performing photoelectron spectroscopy.^30,31^ A potential complication in the detection of Ph^−^ in their setup with a mass resolution of only 1/200 is the fact that copious amounts of dehydrogenated Ph, (Ph-H)^−^, are formed by dissociative electron attachment at a resonance between 7 and 8 eV,^2^ contributing to the mass spectrum, as well as (Ph-H)^−^ ions that contain one ^13^C (its ion yield is 15% of the main isotopologue).

A promising approach to study the properties of a molecule such as Ph with a small or even negative EA is to study complexes of Ph with an adduct M with a vanishingly small EA. The ion-induced dipole interaction between the excess electron and M may increase the EA of MPh^−^ or M_*n*_Ph^−^ clusters, and stabilize the corresponding anion. This approach has been applied by Tschurl *et al.* who reported photoelectron spectra of (H_2_O)_*n*_Ph^−^ with 1 < *n* < 3.^24^ The authors did not observe the bare Ph^−^, but extrapolation to *n* = 0, with correction for the anticipated increase in the solvation shift upon addition of the first solvent water molecule, resulted in an EA of −0.01(4) eV.

Recently, our group studied complexes of Ph with various ligands M with negative (He, H_2_, H_2_O) or extremely small EA (Ca, EA = 24.55 meV) embedded in helium nanodroplets (HNDs).^32^ We showed that long-lived He_*n*_Ph^−^ are formed in HNDs and fragment by low-energy collisions with Ar atoms into He_*n*_Ph^−^ with *n* > 0. Bare Ph^−^ was not observed. Through competition between helium evaporation and electron detachment of He_*n*_Ph^−^ clusters, a lower limit of the vertical detachment energy (VDE) of Ph^−^ of about −3 meV was determined. In case of CaPh^−^ complexes, collision with Ar atoms produces Ca^−^ but no Ph^−^, indicating that the EA of Ph^−^ is below that of Ca, *i.e.* below 24.55 meV.^32^

In this contribution, first, we confirm by performing electron attachment measurements that Ph^−^ is not observed isolated in the gas phase, and secondly, we show that bare Ph^−^ can be stabilized in the HND environment by a suitable choice of parameters, albeit in very small amounts. Nonetheless, from these results we conclude, that Ph has a small, but positive EA.

## Experimental part

Three different experimental setups were used to study the formation of Ph (purchased from Sigma Aldrich, sublimed grade, ≥99.5%) anions. To test the experimental setups, the measurements were repeated with An (purchased from Sigma Aldrich, ReagentPlus, 99%). For all measurements, the sample was vaporized in ultrahigh vacuum at temperatures in the range of 60–70 °C. The recorded mass spectra of anions were analyzed with the software package IsotopeFit, which accounts for isotopic patterns, isobaric ions and the background.^37^

### Resonance electron mass spectrometry

**Fig. 1:**
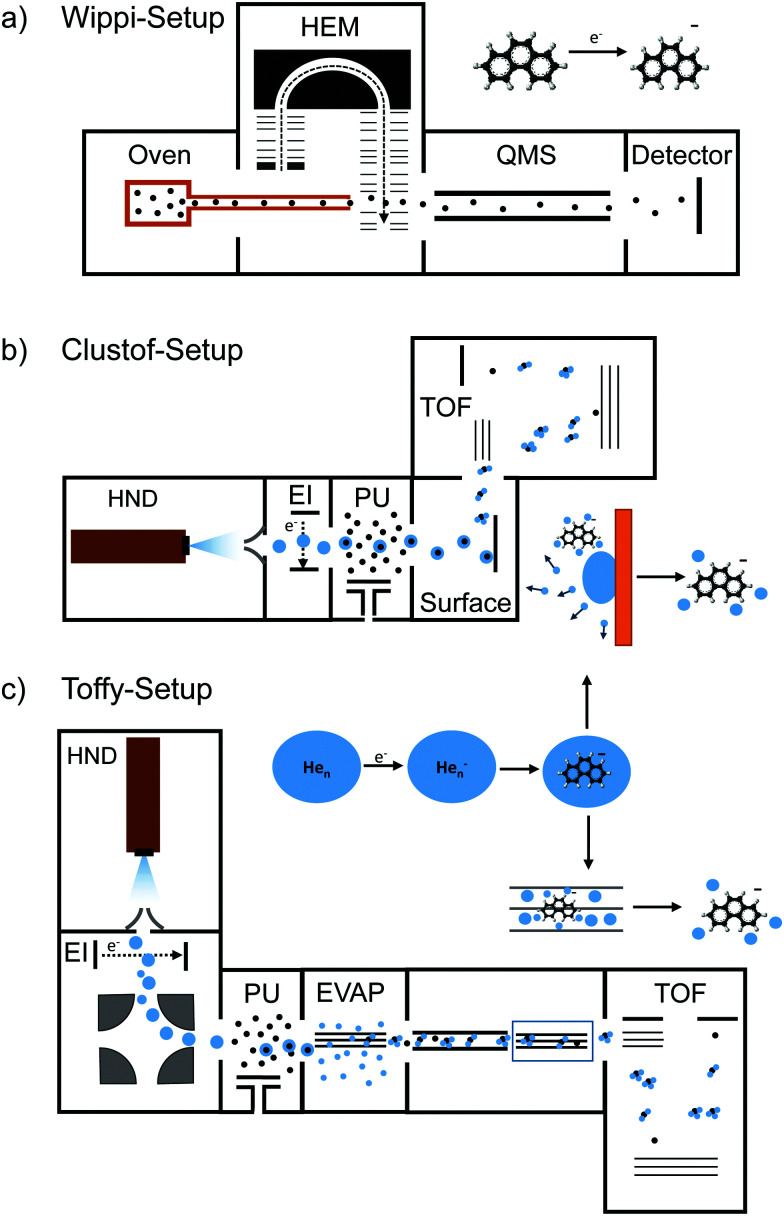
Schematics of the three used experimental setups: (a) Wippi-setup^33,34^ for performing resonance electron mass spectrometry, (b) Clustof-setup^35^ and (c) Toffy-setup^36^ for performing mass spectrometry of ions embedded in HNDs. Details about the setups are described in the main text.

The crossed electron-molecular beam setup Wippi combined with a quadrupole mass spectrometer (QMS), described in detail elsewhere,^33,34^ was used in the present study of electron attachment in the gas phase (see Fig. 1a). An and Ph were vaporized in an oven at 71 and 65 °C, respectively. The vapor entered the interaction chamber of a hemispherical electron monochromator (HEM) through a 1 mm-diameter, stainless-steel and copper capillary, where it crossed an electron beam. The HEM is composed of three parts: a hairpin tungsten filament (heated by applying a current of 2.35 A) used as an electron source, two concentric hemispheres at different electric potentials that function as an energy filter, and two columns forming a series of electrostatic lenses. The latter is used to direct the electron beam from the source to the hemisphere and from the hemisphere to the interaction region. The HEM was tuned to generate the electron beam with an energy resolution of 100 meV (full-width-at-half-maximum, FWHM). The anions were extracted into the quadrupole mass analyzer (QMS) by a weak electrostatic field and detected by a channeltron operated in single ion counting mode. The ion yield of a given mass-selected anion was measured as a function of the incident electron energy. The electron energy scale and energy resolution were determined by measuring the well-known resonances for the formation of SF_6_^−^ from SF_6_ and Cl^−^ from CCl_4_ at 0 eV. The electron current after the interaction region was monitored by using a picoamperemeter.

### Mass spectrometry of PAH ions in anionic HNDs

The mass spectrometric measurements of An and Ph anions embedded in HNDs were performed at two different setups, Clustof^35^ and Toffy.^36^ In both setups, HNDs are produced *via* supersonic expansion by expanding ultrapure helium (Messer, purity 99.9999%) with a stagnation pressure of 28 bar through a 5 μm pinhole nozzle into ultrahigh vacuum. The nozzle is cooled with a closed-cycle cryocooler (Sumitomo Heavy Industries) to 8.8 K. According to Gomez *et al.*^38^ the resulting average droplet size is between 2.5 × 10^5^ and 10^6^ He atoms for the present conditions. To prevent destruction of HNDs by collisions with shock fronts, the resulting jet of He is then passed through a 0.5 mm skimmer (Beam Dynamics, Inc) located about 10 mm after the nozzle, producing a supersonic molecular beam. Afterwards, the HNDs are ionized by passing through a Nier-type electron impact ionization unit, operating at an electron energy of about 22 eV and an electron current of about 200 μA.

In the Clustof setup, the beam of ionized HNDs propagates into the pick-up chamber, where evaporated An or Ph molecules are captured. The doped HNDs collide with an orthogonal metal surface, where the cold droplet “splashes” and evaporates away.^35^ The bare ions with some tens of He atoms attached are extracted and guided towards the time-of-flight (TOF) detector system.

In the Toffy setup, the beam of ionized HNDs is first mass-to-charge selected in a quadrupole bender and then guided into the pickup chamber filled with Ph vapor. In contrast to the Clustof setup, a gentle shrinking of the surrounding He matrix is here enabled by collisions of doped HNDs with He atoms in a subsequent evaporation cell at tunable He pressure. The resulting ionic clusters, optionally decorated with some He atoms, are analyzed in a TOF mass spectrometer (Q-TOF Ultima Waters/Micromass).

## Results

### Electron attachment to bare anthracene or phenanthrene

**Fig. 2:**
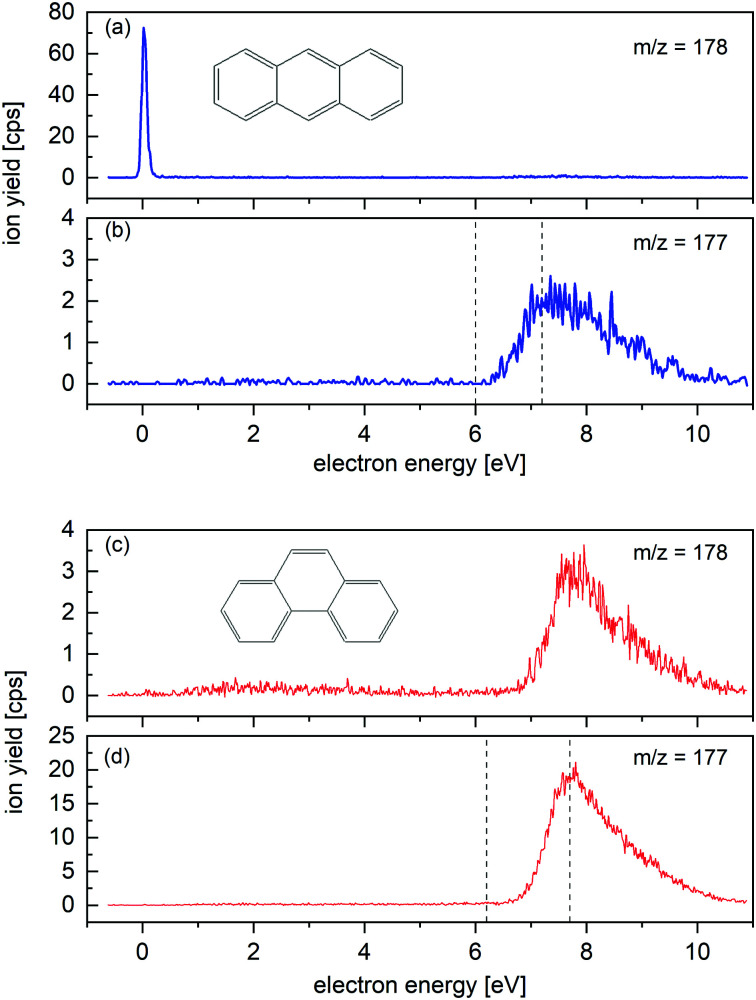
Energy dependence of the ion yield measured for *m*/*z* = 178 and *m*/*z* = 177 upon electron attachment to An (a) and (b) and Ph (c) and (d) in the gas phase. An^−^ parent ions are clearly formed (a), whereas no Ph^−^ parent ions are observed (c). (An-H)^−^ and (Ph-H)^−^ are resonantly formed around 8 eV. Dashed lines in (b) and (d) indicate the onset and the maximum of the yield of dehydrogenated anions reported by Tobita *et al.*^2^ The resonances near 8 eV in (a) and (c) are due to the dehydrogenated anions which contain one ^13^C atom.

Attachment of low-energy electrons to An produces the parent anion An^−^ as shown in Fig. 2a. The very weak resonance which appears in Fig. 2a near 8 eV is not due to An^−^ but to (An-H)^−^ ions that contain one ^13^C atom. The FWHM of the 0 eV resonance is 105 meV, limited by the energy resolution of the primary electron beam. Between 6 and 10 eV dehydrogenated An anions, (An-H)^−^, appear (see Fig. 2b). The resonance was previously observed by Tobita *et al.* who attributed it to a two-particle-one-hole resonance in which capture of the incident electron is accompanied by simultaneous electronic excitation, leading to two electrons in normally unoccupied molecular orbitals.^2^ The onset and the maximum of the resonance reported in their work is indicated by dashed lines; the agreement with our data is excellent.

Attachment of low-energy electrons to Ph does not produce the parent ion, in agreement with the report by Tobita *et al.*^2^ (Ph-H)^−^ is formed resonantly around 8 eV, see Fig. 2d. The onset and maximum of the observed resonance are in good agreement with the values reported by Tobita *et al.* which are indicated in Fig. 2d by dashed lines.^2^ The 8 eV resonance appearing in Fig. 2c is due to (Ph-H)^−^ anions containing one ^13^C isotope, rather than to isotopically pure Ph^−^.

Close inspection of the data in Fig. 2c reveals another broad resonance in the Ph^−^ signal around 3 eV. It is weaker than the 8 eV resonance by a factor of 20. Electron transmission measurements through An reveal two-particle-one-hole resonances in this energy range,^39^ but we are not aware of similar experiments involving Ph. Note that the energy threshold for formation of (Ph-H)^−^ + H is below 3 eV.^2^ The detected signal around 3 eV may correspond to a metastable Ph^−^ parent anion, or to the dissociative electron attachment ion yield from an heavier impurity of the sample. A detectable contamination of the Ph sample with the isomer An is excluded, as no signal is registered at the 0 eV resonance in Fig. 2c.

### Anions extracted from doped HNDs

**Fig. 3:**
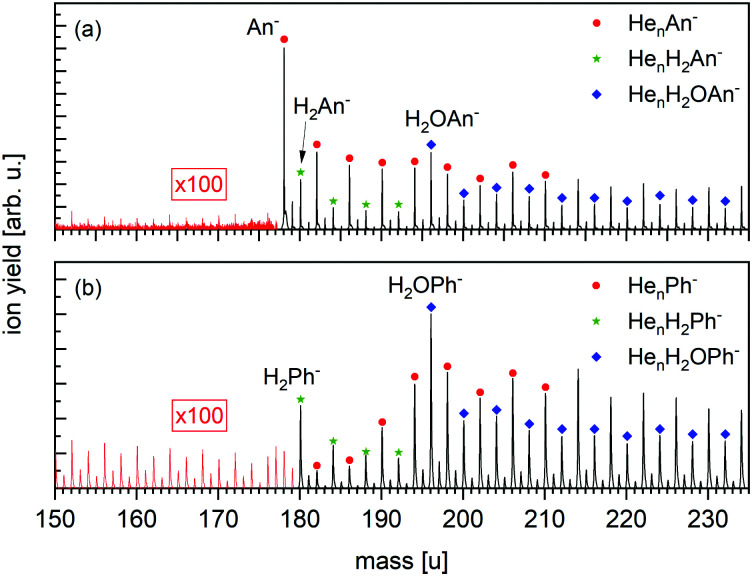
Sections of negative ion mass spectra of HNDs doped with (a) An and (b) Ph measured in the Clustof setup. To increase the visibility of the mass range <180 u, the peaks are multiplied by a factor 100. Labeled mass peaks refer to ions that are isotopically pure (containing ^1^H, ^12^C, ^16^O). While the formation of bare An^−^ is clearly visible in panel (a), the presence of bare Ph^−^ in panel (b) is questionable. For the mass range ≥180 u, anionic complexes of An or Ph with He, or with H_2_ or H_2_O plus He atoms are observed. The detected mass peaks in the mass range <180 u can be linked to He series attached to water clusters.

Fig. 3 displays a negative-ion mass spectrum of HNDs doped with An and Ph, respectively, using the Clustof setup. The strongest mass peak in Fig. 3a is due to An^−^ at mass 178 u, which is to be expected as it is known that An forms long-lived anions upon the capture of low-energy electrons^2,7,40^ and has a positive EA of 0.53 eV. Moreover, three homologous ion series, namely He_*n*_An^−^ (*n* ≥ 0), He_*n*_H_2_An^−^ (*n* ≥ 0) and He_*n*_H_2_OAn^−^ (*n* ≥ 0) are clearly visible in the spectrum. Each of these mass peaks is followed by a satellite peak due to ions that contain one ^13^C (natural abundance 1.07%; their yield is 15% of the main isotopologues). Contributions from ions that contain two ^13^C atoms are negligible (1%). The mass peaks, which are isotopically pure (containing only ^1^H, ^4^He, and ^12^C) are marked. Beginning at mass *m* = 196 u, the He_*n*_H_2_An^−^ (*n* ≥ 4) series cannot be distinguished anymore from the He_*n*_H_2_OAn^−^ (*n* ≥ 0) series. The presence of anions that contain one or more helium atoms attests to their very low vibrational temperature. Water is a contaminant whose appearance cannot be avoided when growing very large HNDs that contain 10^6^ atoms. Moreover, these HNDs have very large collision cross sections and will readily pickup residual gas atoms or molecules in the vacuum system. Likewise, the H_2_ impurities are probably the product of collisions between the HND and residual hydrogen. Turbomolecular pumps that are used in our system have a particularly low compression ratio for hydrogen gas.

The negative ion mass spectrum of Ph in Fig. 3b covers the same mass range as in Fig. 3a. All analogues of ions identified in Fig. 3a are observed, namely He_*n*_Ph^−^ (*n* ≥ 1), He_*n*_H_2_Ph^−^ (*n* ≥ 0) and He_*n*_H_2_OPh^−^ (*n* ≥ 0), except for the absence of the bare parent ion Ph^−^ which would appear at 178 u.

**Fig. 4:**
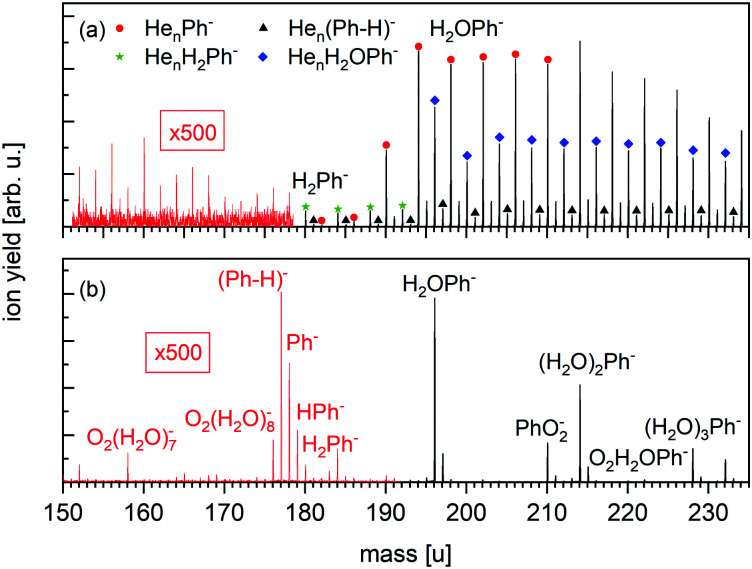
Sections of negative ion mass spectra of HNDs doped with Ph measured in the Toffy setup for (a) *P*_Evap_ = 0.1 Pa and (b) *P*_Evap_ = 0.2 Pa (the measured pressure was corrected by taking the gas correction factor of He into account). (a) Anionic complexes of Ph with He, or with H_2_ or H_2_O plus He atoms are observed. Moreover, a small contribution of dehydrogenated He_*n*_(Ph-H)^−^ (*n* ≥ 1) is detected. (b) A complete removal of the He matrix by collisions with He atoms in the evaporation chamber shows that a small amount of bare Ph^−^ and dehydrogenated (Ph-H)^−^ is stabilized in the HND environment and survives for several ms.

**Fig. 5:**
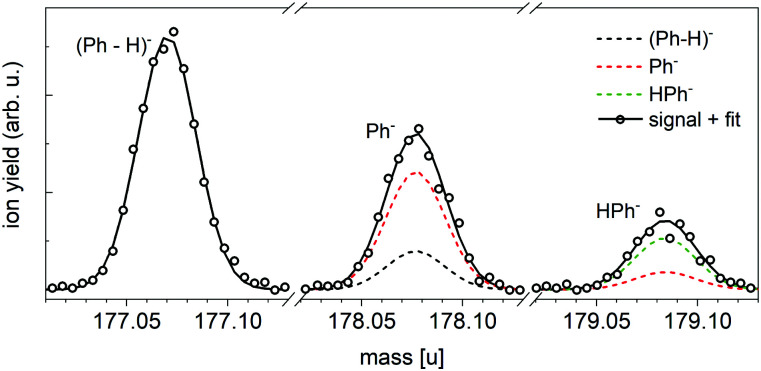
Expanded view of the anion mass spectrum of Fig. 4b recorded at *P*_Evap_ = 0.2 Pa. Dehydrogenated Ph anions (Ph-H)^−^ as well as bare Ph^−^ and HPh^−^ are visible. The dashed lines show the contribution of these ions under consideration of the minor isotopes with one ^13^C. 15% of the measured ion yield at mass 178 u arises from the dehydrogenated anion (Ph-H)^−^ that contains one ^13^C, and 15% of the measured ion yield at mass 179 u arises from the bare Ph anion that contains one ^13^C. The sum of all isotopic contributions results into the solid line, which fits the measured ion yield.

The negative ion mass spectrum of Ph in Fig. 4 was obtained by using the Toffy setup. While the ionization and doping of the HNDs proceed in the same way as in Clustof, the dopant ions are made accessible for mass spectrometry in a different way. Instead of being collided with a surface, the He matrix is softly removed by collisions with He atoms. The tuning of the He pressure in the evaporation chamber enables to control the size of the He matrix, in which the dopant ions are embedded. Fig. 4a shows the mass spectrum for an evaporation pressure of 0.1 Pa and Fig. 4b for an evaporation pressure of 0.2 Pa (the measured pressure was corrected by taking the gas correction factor of He into account). Fig. 4a looks similar to the mass spectrum of Fig. 3a obtained with the Clustof setup. The dominant peaks can be linked to He_*n*_Ph^−^ (*n* ≥ 1), He_*n*_(Ph-H)^−^ (*n* ≥ 1), He_*n*_H_2_Ph^−^ (*n* ≥ 0), and He_*n*_H_2_OPh^−^ (*n* ≥ 0). A zoom-in of the lower mass range <180 u (ion yield multiplied by a factor of 500 to increase the visibility of the mass peaks), does not show a clear signal of the bare parent ion Ph^−^ here either. The observed peaks can be linked to He series attached to impurities. The situation changes when the He matrix is further reduced (Fig. 4b). Again, the most prominent peaks are due to clustering of Ph with impurities (H_2_O, O_2_, H_2_) followed by a satellite peak due to isotopologues that contain one ^13^C. Nevertheless, a closer look at masses <180 u (the ion yield is multiplied again by a factor of 500 to increase the visibility of the mass peaks), shows not only a weak mass peak at 177 u due to dehydrogenated Ph anions, (Ph-H)^−^, but also another peak at 178 u which is more intense than the expected contribution from the ^13^C-containing isotopologue of (Ph-H)^−^. For further inspection, Fig. 5 shows a zoom-in of this mass section with the expected isotopic patterns. 15% of the measured ion yield at mass 178 u (the mass of the parent Ph anion) arises from the dehydrogenated anion (Ph-H)^−^ that contains one ^13^C and 15% of the measured ion yield at mass 179 u arises from the bare Ph anion, contributing to the protonated Ph peak. In contrast to the stabilization of the transient molecule SF_6_^+^ in HNDs by the formation of SF_5_^+^F clusters,^41,42^ in the present case, the ultracold HND environment enables the stabilization of bare as well as dehydrogenated Ph anions.

## Discussion

While for the resonant electron-attachment measurements in the gas phase at room temperature no Ph^−^ was detected, a small amount of the latter became visible in the HND environment, after gently removing the surrounding He matrix by collisions with He atoms. Each collision will transfer, on average, 0.05 eV to the doped HND, ∼80 times the evaporation energy of bulk helium, leading to partial or complete evaporation of the He matrix. By selecting suitable parameters for the evaporation pressure, Ph^−^ becomes detectable. Under the assumption that the bare Ph anion is born in the evaporation chamber, a minimum lifetime of several ms is necessary to detect the anion in the TOF. In previously performed collision induced dissociation (CID) measurements we collided Ar atoms with He_*n*_Ph^−^ (8 ≥ *n* > 0) complexes. Only He_*n*_Ph^−^ (7 ≥ *n* > 0) fragments, but no bare Ph^−^ were detected.^32^ The “non-detection” of the bare anion might be explainable by the higher transferred collision energy of Ar atoms in comparison to He atoms. Moreover, the measurements were aggravated by larger noise/poorer counting statistics.

An even more unexpected observation of the measurements presented in Fig. 4 is the formation of He_*n*_(Ph-H)^−^ in the HND environment. We know from gas-phase electron-attachment measurements, that an electron energy of 7–8 eV is necessary to cause dehydrogenation. To retrace the underlying process inside the HND, we have to revisit the formation of HND anions and charge transfer to the embedded dopants.^43^

The formation of negatively charged HNDs can proceed *via* two mechanisms. In one case, low energy electrons of about 2 eV overcome the HND surface barrier (between 0.6 and 1.1 eV), will thermalize and be trapped in the HND forming a so-called electron bubble, a void with a radius of 1.7 nm. In the second case, penetrating electrons at around 22 eV may lead to the excitation of a helium atom (the first three excited states are at 19.8 eV, 20.9 eV and at 22.7 eV) and subsequent capture of the scattered incident electron. The process terminates in the formation of He*^−^, which is solvated in the droplet and may interact with another helium atom to form He_2_*^−^.^44^ In the following, He*^−^ (or He_2_*^−^) may de-excite into the ground state, transferring the released energy to the ‘attached’ electron. Another possible, but less probable de-excitation path is the interaction of He*^−^ or He_2_*^−^ with a dopant M or another excited He atom, leading to Penning ionization He*^−^ + M → He + M^+^ + 2e^−^ or He*^−^ + He* → He + He^+^ + 2e^−^, delivering two free electrons. The electrons from these de-excitation channels may eventually interact with the dopants while moving through the HND. Investigations on Penning ionization of acene molecules in EUV-excited HNDs have shown a broad spectrum of kinetic energies of the Penning electron, indicating that the emitted electrons are severely affected by collective electron helium interactions.^45^ Thus, electrons of a broad range of kinetic energies are formed and may interact with the dopants. The parent anion Ph^−^ as well as the dehydrogenated anion (Ph-H)^−^ may be formed by this sequence of events.

As long as the dopant anions are embedded in a He matrix, the formation of He_*n*_Ph^−^ dominates over the formation of the He_*n*_(Ph-H)^−^ channel. This is nicely observed in Fig. 4a and also in Fig. 3, even though the water and hydrogen impurities are the more dominating contributions. The He matrix seems to efficiently quench the hydrogen-loss channel, the only channel observed for electron attachment to bare Ph in the gas phase (see Fig. 2c and d). A complete evaporation of the helium matrix means the removal of the fragmentation quencher, and the formation of dehydrogenated anions (Ph-H)^−^ dominates over the stabilization of the bare parent anion Ph^−^, as seen in Fig. 4b and 5.

## Conclusion

In this contribution, we have confirmed the non-detection of Ph^−^ in the gas-phase after binary collision with an electron, and the stabilization of the latter in the HND environment, even though the measured ion yield was minor in comparison to Ph anions complexed with other atoms or molecules. From these and previous observations we conclude, that Ph has a small (<24.55 meV), but positive EA. Due to the small electron affinity, it can be expected that bare Ph anions are hardly found in interstellar clouds. However, complexation of Ph with other molecules like H_2_ and H_2_O as well as the clustering to larger Ph complexes (dimers, trimers, *etc.*) leads to a considerable increase of the electron affinity. These complexes might be abundantly present in the interstellar medium and play an important role for the interstellar chemistry.

Besides bare Ph anions, we also detected dehydrogenated Ph anions (Ph-H)^−^ in the HND environment. The formation of the latter was rationalized by retracing the formation of HND anions, the de-excitation paths of excited He*^−^ and He_2_*^−^ as well as associated charge transfer processes to the embedded dopants. Once more, it has been shown that helium nanodroplets generate a versatile and suitable environment to stabilize and study metastable molecular ions.

## Conflicts of interest

The authors declare no conflict of interest.

## Supplementary Material

## References

[cit1] Wakelam V., Herbst E. (2008). Astrophys. J..

[cit2] Tobita S., Meinke M., Illenberger E., Christophorou L. G., Baumgärtel H., Leach S. (1992). Chem. Phys..

[cit3] Betowski L. D., Enlow M., Aue D. H. (2006). Int. J. Mass Spectrom..

[cit4] Khatymov R. V., Tuktarov R. F., Muftakhov M. V. (2011). JETP Lett..

[cit5] Khatymov R. V., Muftakhov M. V., Shchukin P. V. (2017). Rapid Commun. Mass Spectrom..

[cit6] Kregel S. J., Thurston G. K., Garand E. (2018). J. Chem. Phys..

[cit7] Schiedt J., Weinkauf R. (1997). Chem. Phys. Lett..

[cit8] Song J. K., Lee N. K., Kim J. H., Han S. Y., Kim S. K. (2003). J. Chem. Phys..

[cit9] Ando N., Mitsui M., Nakajima A. (2007). J. Chem. Phys..

[cit10] Becker R. S., Chen E. (1966). J. Chem. Phys..

[cit11] Wojnárovits L., Földiák G. (1981). J. Chromatogr. A.

[cit12] Chen G., Cooks R. G. (1995). J. Mass Spectrom..

[cit13] Malloci G., Mulas G., Cappellini G., Fiorentini V., Porceddu I. (2005). Astron. Astrophys..

[cit14] Betowski L., Enlow M., Aue D. H. (2006). Int. J. Mass Spectrom..

[cit15] Betowski L. D., Enlow M., Riddick L., Aue D. H. (2006). J. Phys. Chem. A.

[cit16] Kadantsev E. S., Stott M., Rubio A. (2006). J. Chem. Phys..

[cit17] Modelli A., Mussoni L. (2007). Chem. Phys..

[cit18] Kukhta A., Kukhta I., Kukhta N., Neyra O., Meza E. (2008). J. Phys. B.

[cit19] Carelli F., Gianturco F. A., Satta M., Sebastianelli F. (2014). Int. J. Mass Spectrom..

[cit20] Richard R. M., Marshall M. S., Dolgounitcheva O., Ortiz J. V., Bredas J.-L., Marom N., Sherrill C. D. (2016). J. Chem. Theory Comput..

[cit21] Hajgató B., Deleuze M., Tozer D., De Proft F. (2008). J. Chem. Phys..

[cit22] Steinfelder K., Tümmler R. (1961). Angew. Chem..

[cit23] Lietard A., Mensa-Bonsu G., Verlet J. R. R. (2021). Nat. Chem..

[cit24] Tschurl M., Boesl U., Gilb S. (2006). J. Chem. Phys..

[cit25] Chen E., Wentworth W. (1989). Mol. Cryst. Liq. Cryst..

[cit26] Ruoff R. S., Kadish K. M., Boulas P., Chen E. (1995). J. Phys. Chem..

[cit27] Chen E. C., Chen E. S. (2018). J. Chromatogr. A.

[cit28] Ervin K. M., Anusiewicz I., Skurski P., Simons J., Lineberger W. C. (2003). J. Phys. Chem. A.

[cit29] Huzak M., Hajgató B., Deleuze M. (2012). Chem. Phys..

[cit30] Lee S. H., Kim N., Ha D. G., Song J. K. (2013). RSC Adv..

[cit31] Lee S. H., Song J. K., Kim S. K. (2015). Chem. Phys. Lett..

[cit32] Gruber E., Kollotzek S., Bergmeister S., Zappa F., Ončák M., Scheier P., Echt O. (2022). Phys. Chem. Chem. Phys..

[cit33] Denifl S., Ptasinska S., Sonnweber B., Scheier P., Liu D., Hagelberg F., Mack J., Scott L. T., Mark T. D. (2005). J. Chem. Phys..

[cit34] Mahmoodi-Darian M., Mauracher A., Aleem A., Denifl S., Rittenschober B., Bacher A., Probst M., Mark T., Scheier P. (2009). J. Phys. Chem. A.

[cit35] Martini P., Albertini S., Laimer F., Meyer M., Gatchell M., Echt O., Zappa F., Scheier P. (2021). Phys. Rev. Lett..

[cit36] Tiefenthaler L., Ameixa J., Martini P., Albertini S., Ballauf L., Zankl M., Goulart M., Laimer F., von Haeften K., Zappa F., Scheier P. (2020). Rev. Sci. Instrum..

[cit37] Ralser S., Postler J., Harnisch M., Ellis A. M., Scheier P. (2015). Int. J. Mass Spectrom..

[cit38] Gomez L. F., Loginov E., Sliter R., Vilesov A. F. (2011). J. Chem. Phys..

[cit39] Burrow P., Michejda J., Jordan K. (1987). J. Chem. Phys..

[cit40] Ardenne M. V., Steinfelder K., Tummler R. (1961). Angew. Chem..

[cit41] Albertini S., Bergmeister S., Laimer F., Martini P., Gruber E., Zappa F., OncÌŒák M., Scheier P., Echt O. (2021). J. Phys. Chem. Lett..

[cit42] Zunzunegui-Bru E., Gruber E., Bergmeister S., Meyer M., Zappa F., Bartolomei M., Pirani F., Villarreal P., González-Lezana T., Scheier P. (2022). Phys. Chem. Chem. Phys..

[cit43] Mauracher A., Echt O., Ellis A., Yang S., Bohme D., Postler J., Kaiser A., Denifl S., Scheier P. (2018). Phys. Rep..

[cit44] Albertini S., Gruber E., Zappa F., Krasnokutski S., Laimer F., Scheier P. (2021). Mass Spectrom. Rev..

[cit45] Shcherbinin M., LaForge A., Hanif M., Richter R., Mudrich M. (2018). J. Phys. Chem. A.

